# ZIF-8 derived Ag-doped ZnO photocatalyst with enhanced photocatalytic activity

**DOI:** 10.1039/c7ra13351k

**Published:** 2018-01-29

**Authors:** Xiaobing Yang, Liqing Qiu, Xuetao Luo

**Affiliations:** College of Ecology and Resource Engineering, Wuyi University Fujian Wuyishan 354300 China; Fujian Provincial Key Laboratory of Eco-Industrial Green Technology, Wuyi University Fujian Wuyishan 354300 China; Fujian Key Laboratory of Advanced Materials, College of Materials, Xiamen University Xiamen 361005 China xuetao@xmu.edu.cn

## Abstract

Recently, zeolitic imidazolate framework-8 (ZIF-8) has been widely studied and used as a catalyst in various fields, due to its high specific surface area, tunable channels and thermal and chemical stability. In this paper, ZIF-8 was used as a precursor to fabricate a Ag/ZnO photocatalyst, and the influence of Ag on the photocatalytic activity of ZnO has been explored. All samples were characterised using XRD, SEM, TEM, and UV-vis diffuse reflectance spectra. The photocatalytic activity of all samples was evaluated by the degradation of a rhodamine B solution under UV light. The results show that ZIF-8 was completely transformed into ZnO when it was calcined at 550 °C for 6 h, and Ag was well loaded onto ZnO. The photocatalytic efficiency of ZnO is 92.32%. When ZnO was doped with Ag, its photocatalytic efficiency was highly improved (99.64%). Furthermore, Ag/ZnO exhibited high photocatalytic stability. After five repeated cycles, the photocatalytic activity of Ag/ZnO was highly retained at 97.48%.

## Abstract 2

In this paper, ZIF-8 was used as a precursor to fabricate a Ag/ZnO photocatalyst, and the influence of Ag on the photocatalytic activity of ZnO has also been explored.

## Graphical abstract

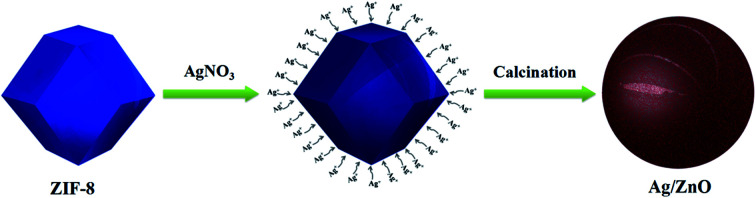

## 1. Introduction

To date, photocatalytic degradation has been one effective way of treating various water pollutants, and semiconductor photocatalysts have been widely investigated. Over several decades of studies, many semiconductor photocatalysts have been found, such as TiO_2_,^1,2^ SnO_2_,^3^ ZrO_2_,^4^ and ZnO.^5^ They possess a high degradation capacity toward toxic and recalcitrant chemical species through relatively simple and low-cost procedures. Among these semiconductor photocatalysts, ZnO, with a band gap of 3.2 eV, has been widely investigated, due to its powerful oxidation capability, non-toxicity and chemical stability.^6^ It can degrade organic dyes into non-toxic substances.

In order to expand the usage of ZnO, researchers have made efforts to improve its catalytic efficiency. One of the main problems affecting the deactivation of ZnO is the high tendency of the hole–electron pair to recombine, so it is a challenge to ensure the effective separation of the electron–hole pairs of ZnO. After a few decades of research, researchers have found that doping with some metals, such as manganese, cobalt, silver, and so on, is a good strategy to overcome this problem. For instance, Ullah *et al.* used wet-chemical techniques to prepare the Mn-doped ZnO, and found that the photo-degradation efficiency of Mn-doped ZnO was significantly higher than that of the undoped ZnO.^7^ Xu *et al.* adopted a hydrothermal method to prepare ZnO powders with different Co^2+^ doping concentrations (0, 0.5, 1, 3, and 5 mol%).^8^ When the doping concentration was 3 mol%, the degradation ratio of MO reached 78% after reaction for 240 min. Divband *et al.* employed photo reduction, chemical reduction and polyacrylamide-gel methods to obtain Ag/ZnO photocatalysts with different Ag loadings.^9^ They found that the metallic Ag in the Ag/ZnO photocatalysts facilitated the trapping of photogenerated electrons and improved the photocatalytic activity of the Ag/ZnO photocatalysts. Ag/ZnO prepared by the polyacrylamide gel method exhibited the best photocatalytic performance, in comparison with that prepared by the chemical reduction and photo reduction methods.

As a kind of MOF, ZIF-8 has a high specific surface area and tunable channels, as well as thermal and chemical stability.^10–12^ Due to its excellent properties, it has been widely used in various catalytic fields such as CO oxidation,^13^ Friedel–Crafts acylation,^14^ and cyclohexene hydrogenation.^15^ ZIF-8 is constructed from Zn(ii) and 2-methylimidazole ligands. So, ZIF-8 is expected to be used as a Zn source to prepare ZnO. In this paper, we adopted a facile method that combines an adsorption method with heat treatment to prepare Ag doped ZnO by using ZIF-8 as a precursor. We explored the influence of Ag on the properties of the ZnO derived from ZIF-8. The photocatalytic activity of all samples was evaluated by the degradation of rhodamine B in aqueous solution under UV light. After careful studying, Ag/ZnO shows higher photocatalytic activity than the pure ZnO derived from ZIF-8. We expect that Ag/ZnO can be used as a photocatalyst for the degradation of some organic dyes coming from contaminants.

## 2. Experimental

### 2.1 Chemicals

Zinc nitrate hexahydrate [Zn(NO_3_)_2_·6H_2_O, ≥98%], methanol (≥99%), ethyl alcohol (≥99%), rhodamine B (RhB) and silver nitrate (AgNO_3_, ≥99.8%) were purchased from Sinopharm Chemical Reagent Co. Ltd. 2-Methylimidazole (Hmim, ≥99%) was obtained from Chengdu Kelong Chemical Reagent Company. All chemicals were used directly without any further purification. Distilled water was used throughout the experiments.

### 2.2 Fabrication of the Ag doped ZnO photocatalyst

The Ag doped ZnO photocatalyst was derived from ZIF-8. The procedures include the preparation of ZIF-8 and the fabrication of the Ag doped ZnO photocatalyst.

ZIF-8 was prepared using our previous research method. Firstly, 10 mmol of Zn(NO_3_)_2_·6H_2_O was dissolved in 100 mL of methanol to form the A solution, and 40 mmol of 2-methylimidazole was added into 100 mL of methanol to form the B solution. Then, the A solution was slowly poured into the B solution with continuous stirring. After five minutes of stirring, the mixture was kept standing at room temperature for 24 h. The mixture was centrifuged and dried at 70 °C to obtain ZIF-8.

**Fig. 1:**
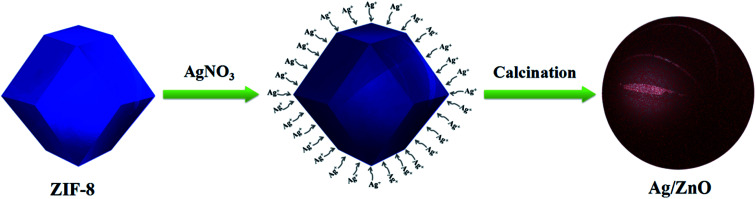
The synthetic procedure for the Ag/ZnO photocatalyst.

Ag doped ZnO (Ag/ZnO) was prepared from ZIF-8 by the following method: firstly, 0.5 mM AgNO_3_ was dissolved in 20 mL of ethanol containing 2 mL of distilled water to form the AgNO_3_ solution. Then, 0.5 g of ZIF-8 was dispersed in the AgNO_3_ solution with vigorous stirring for 60 min. After that, the AgNO_3_ treated ZIF-8 was washed with absolute ethanol to remove the residual Ag^+^ adsorbed on the surface of the ZIF-8 nanoparticles, and dried at 50 °C. Finally, the Ag doped ZIF-8 was calcined at 550 °C for 6 h, to remove the organic agents and obtain the Ag/ZnO. The synthetic procedure for the Ag/ZnO photocatalyst is shown in Fig. 1.

### 2.3 Characterization of the products

X-ray powder diffraction (XRD) data were recorded on a Bruker-AxsD8 diffractometer using Cu-Kα radiation in the angular range (2*θ*) from 5 to 60°, operated at 40 kV and 40 mA. Scanning electron microscope (SEM) images were directly observed using a Hitachi SU70 scanning microscope at an accelerating voltage of 5 kV. Electron transmission microscopy (TEM) images were obtained using an F30 under an accelerating voltage of 100 kV. UV-vis absorption spectra were measured on a UV-101 PC scanning spectrophotometer.

### 2.4 Catalytic activity testing

Rhodamine B (RhB) was used as a representative dye to test the photocatalytic activity of all the samples under UV light. Firstly, 0.5 g of the photocatalyst was dispersed in 100 mL of a nicotine solution (2 × 10^−2^ g L^−1^), and kept stirring for 30 min in the dark to reach an adsorption–desorption equilibrium between RhB and the photocatalyst. Then, a 300 W lamp immobilized on top of the reactor was turned on (the distance between the lamp and reactor was 10 cm). At regular intervals during this photocatalytic process, several milliliters of the suspension solution were withdrawn from the reactor, centrifuged to remove the catalyst, and the RhB concentration in solution was measured using a UV-vis spectrophotometer.

## 3. Results and discussion

**Fig. 2:**
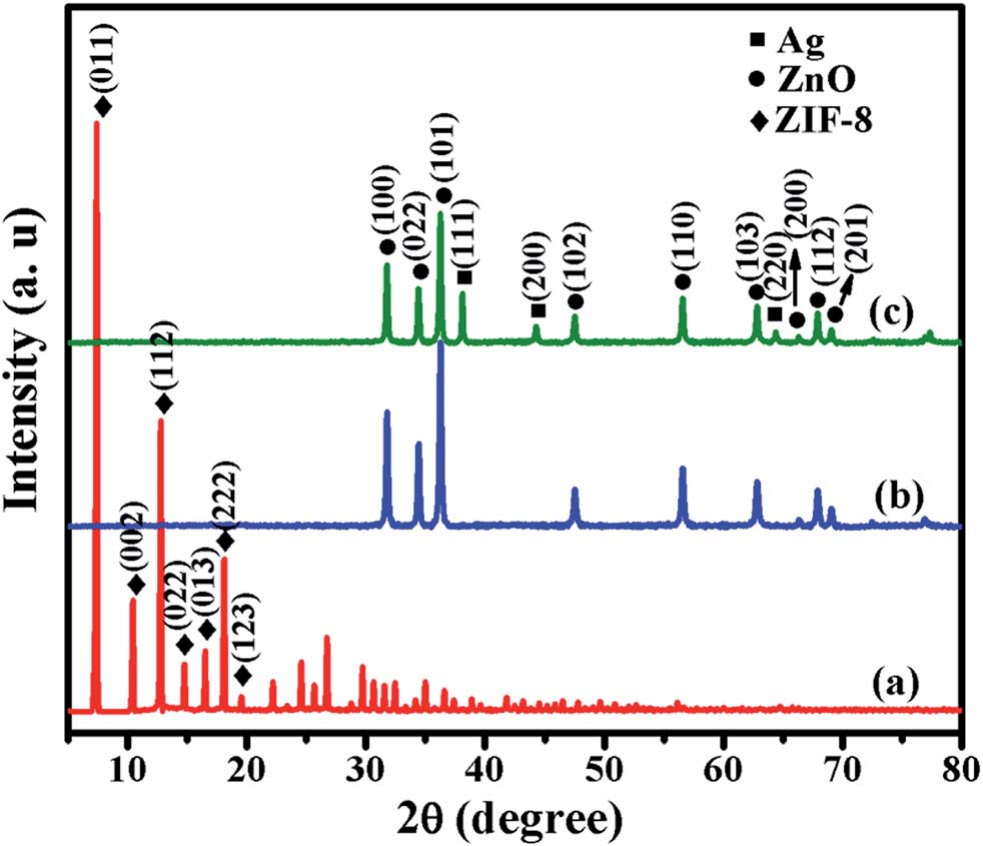
XRD patterns of (a) ZIF-8, (b) ZnO, and (c) Ag/ZnO.

The X-ray diffraction (XRD) patterns of ZIF-8, ZnO and Ag/ZnO are shown in Fig. 2. Fig. 2a shows distinct peaks at 7.38°, 10.42°, 12.77°, 14.75°, 16.50°, 18.08° and 19.53°, which are ascribable to the (011), (002), (112), (022), (013), (222) and (123) reflections of ZIF-8, respectively.^16^ When ZIF-8 is calcined at 550 °C for 6 h, the original peaks corresponding to ZIF-8 are no longer present. However, it exhibits new peaks at 31.76°, 34.44°, 36.25°, 47.54°, 56.58°, 62.85°, 66.35°, 67.93° and 69.08° (Fig. 2b), which are ascribable to the (100), (022), (101), (102), (110), (103), (200), (112) and (201) reflections of ZnO, respectively.^17^Fig. 2c shows the XRD pattern of Ag/ZnO. In addition to the diffraction peaks of ZnO, it shows new peaks at 38.10°, 44.29° and 64.41°, which are ascribable to the (111), (200) and (220) reflections of Ag, respectively.^18^ These results indicate that ZIF-8 is absolutely transformed into ZnO, and that Ag is well loaded onto the ZnO.

**Fig. 3:**
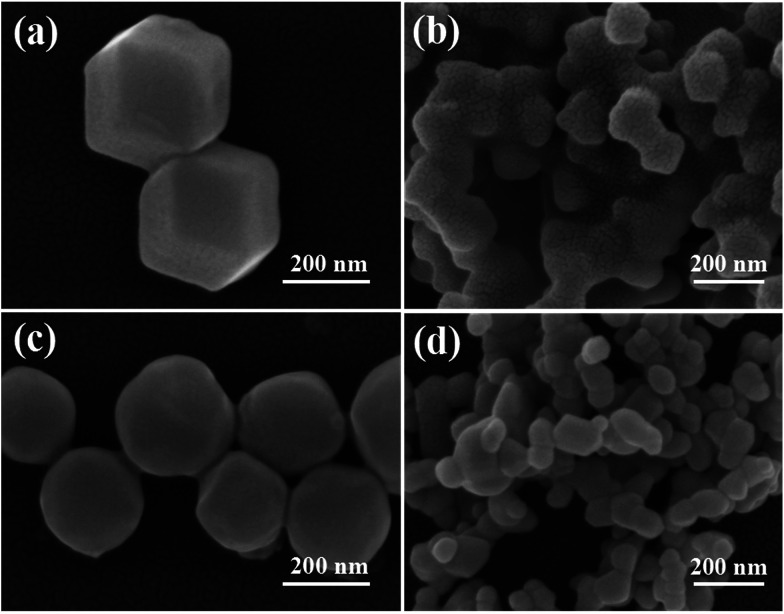
SEM images of (a) ZIF-8, (b) ZnO, (c) ZIF-8 treated with AgNO_3_, and (d) Ag/ZnO.

The morphology of all the samples was obtained using a scanning electron microscope, and the results are shown in Fig. 3. Fig. 3a shows the SEM image of ZIF-8, which exhibits a dodecahedral morphology. When ZIF-8 is calcined at 550 °C for 6 h, the structure is collapsed (Fig. 3b), and the particle size decreases from 300 nm to 100 nm. When ZIF-8 is treated with AgNO_3_, its edges become rounded (Fig. 3c), which is consistent with Wee’s report.^16^Fig. 3d shows the SEM image of Ag/ZnO. The particles of ZIF-8, treated with AgNO_3_, are also collapsed.

**Fig. 4:**
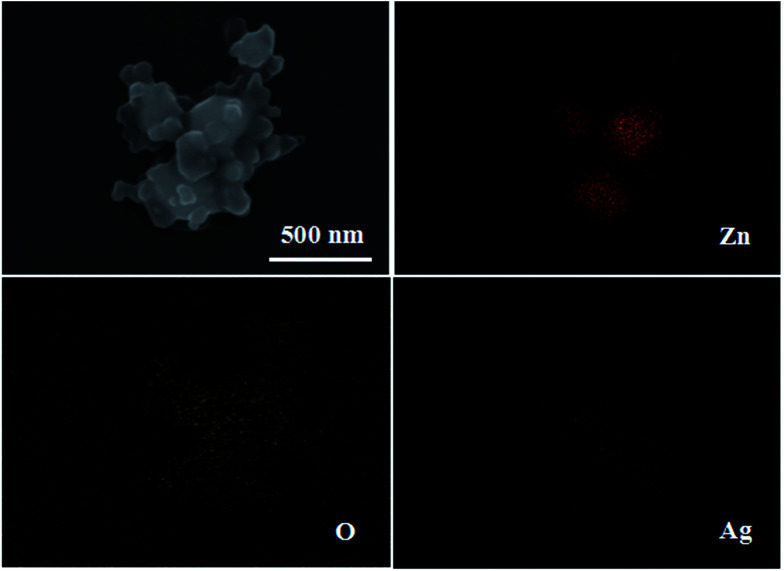
The EDS maps of Ag/ZnO.

Fig. 4 shows the EDS maps of Ag/ZnO; the EDS images confirm the presence of the elements Zn, O and Ag. Ag is well loaded and dispersed in ZnO nanoparticles. This indicates that the organic ligands of ZIF-8 are absolutely removed when it is calcined at 550 °C for 6 h.

**Fig. 5:**
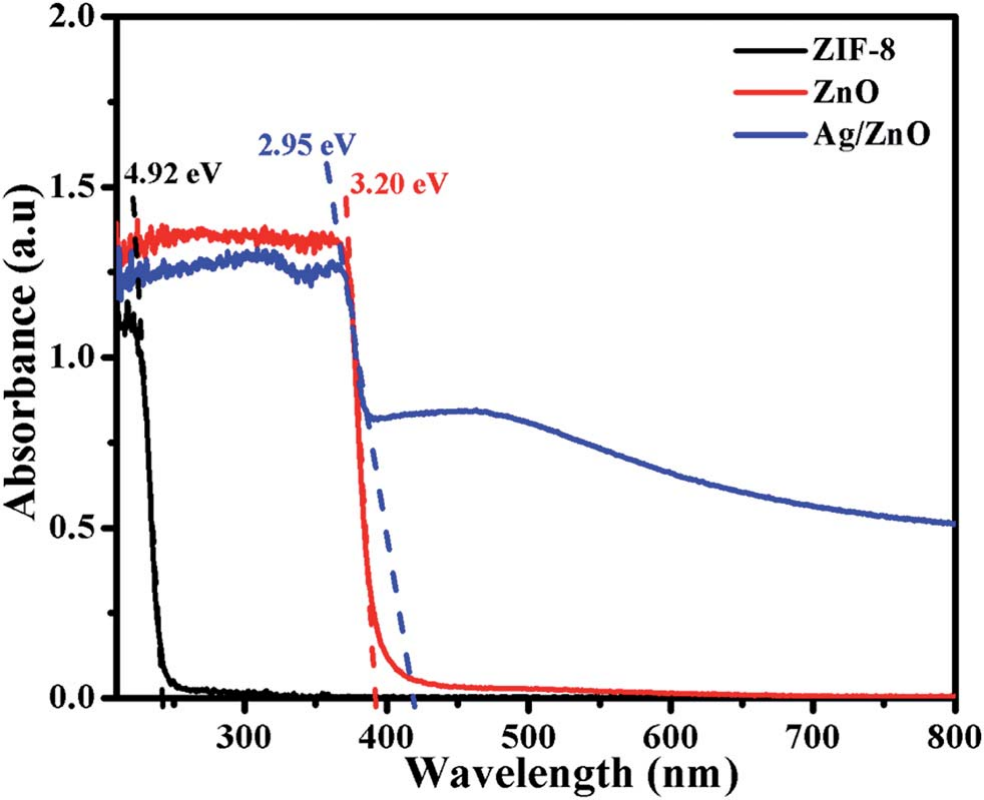
UV-vis spectra of ZIF-8, ZnO, and Ag/ZnO.

Fig. 5 shows the UV-vis absorption spectra of ZIF-8, ZnO and Ag/ZnO. The spectrum of ZIF-8 shows a sharp peak at 252 nm, which is attributed to the excitonic absorption of ZIF-8. When ZIF-8 is calcined at 550 °C for 6 h, it exhibits a strong absorption peak at 388 nm. This indicates that ZIF-8 is completely transformed into ZnO, which is consistent with the XRD patterns (Fig. 2b). When ZIF-8 is treated with AgNO_3_ and calcined at 550 °C for 6 h, its characteristic absorption, corresponding to ZnO, exhibits a red shift to 420 nm. Compared to ZnO, the Ag doped ZnO (Ag/ZnO) has a broad absorption in the range of 400–600 nm, which is probably caused by the strong interfacial coupling between the neighbouring ZnO and Ag NPs.^17^ The band gap of the sample can be obtained by the following formula:^18^

1 *A* = [*k*(*hν* − *E*_g_)1/2]/*hν*

where *A* is the absorbance, *k* is a constant, *h* is the Planck’s constant, *ν* is the frequency of light, and *E*_g_ is the band gap of the sample. According to the results shown in Fig. 5, the band gaps of ZIF-8 and ZnO are 4.92 eV and 3.20 eV, respectively. Ag/ZnO has a band gap of 2.95 eV, and has a broad band gap in the range of 3.10–2.07 eV.

**Fig. 6:**
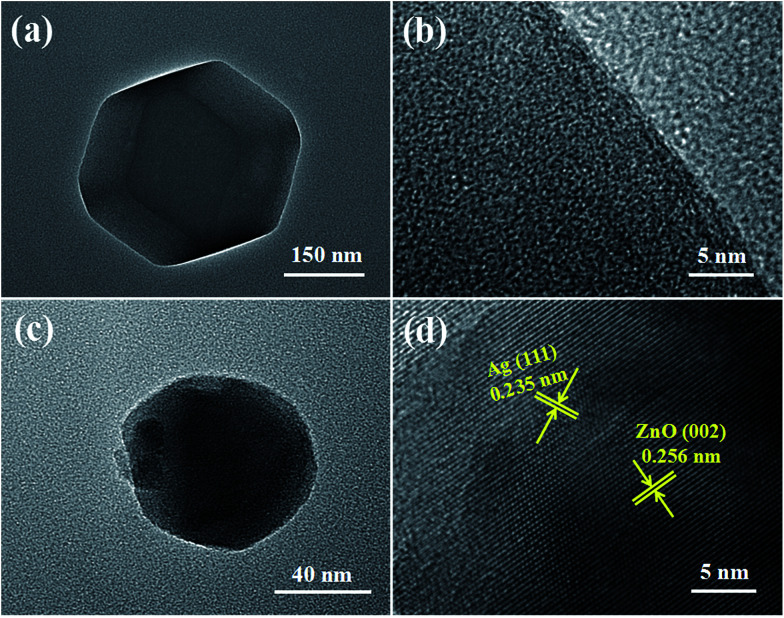
Low magnification TEM images and HRTEM images of (a and b) ZIF-8 and (c and d) Ag/ZnO.

The surface morphologies of ZIF-8 and Ag/ZnO were analyzed using low magnification TEM images and HRTEM images. Fig. 6a shows the low magnification TEM image of ZIF-8. ZIF-8 displays a dodecahedral morphology. Fig. 6b shows the HRTEM image of ZIF-8. ZIF-8 displays a mesoporous structure, which is in accordance with Pan’s report.^19^Fig. 6c shows the low magnification TEM image of Ag/ZnO; the particle size of Ag/ZnO is about 100 nm. The high-resolution TEM (HRTEM) (Fig. 6d) of the region shows the characteristic lattice fringes of 0.235 nm and 0.256 nm, which are assigned to the (111) plane of Ag and (002) plane of ZnO, respectively. The Ag nanoparticles are well dispersed in ZnO.

**Fig. 7:**
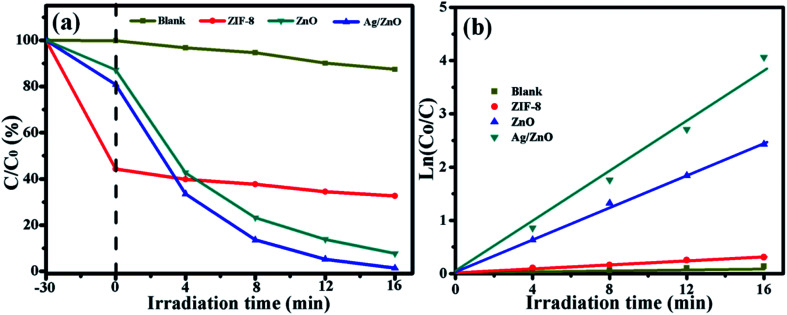
The photocatalytic degradation (a) and the kinetics (b) of blank, ZIF-8, ZnO, and Ag/ZnO, for the absorption of RhB in the dark and degradation of RhB under UV light irradiation.

The photocatalytic activity of all the photocatalysts was evaluated by the degradation of a RhB solution, and the results are shown in Fig. 7. Fig. 7a shows the photocatalytic degradation of RhB by all the photocatalysts under UV light. In order to eliminate the influence of the adsorption of the photocatalyst, every experiment was carried out in the dark for 30 min. After 30 min of dark reaction, 55.67% of RhB was adsorbed by ZIF-8, and 17.12% and 16.43% of RhB were adsorbed by ZnO and Ag/ZnO, respectively. Then, they were exposed to UV light. After 16 min of irradiation, 12.57% of RhB was self-degraded. ZIF-8 showed almost no degradation properties, ZnO could degrade 92.32% of RhB, and the photocatalytic efficiency of Ag/ZnO was 99.64%. Doping with Ag could highly increase the photocatalytic activity of ZnO. Fig. 7b shows the kinetics of all the photocatalysts for the degradation of RhB. The photocatalytic degradation kinetic reaction can be described by ln(*C*_0_/*C*) = *kt* (where *k* is a pseudo-first-rate kinetic constant and *t* is the irradiation time). The calculated *k* value of ZnO is 0.154 min^−1^, but the *k* value of Ag/ZnO is 0.241 min^−1^, which is 1.56 times higher than that of ZnO; Ag/ZnO shows the highest kinetic constant.

**Fig. 8:**
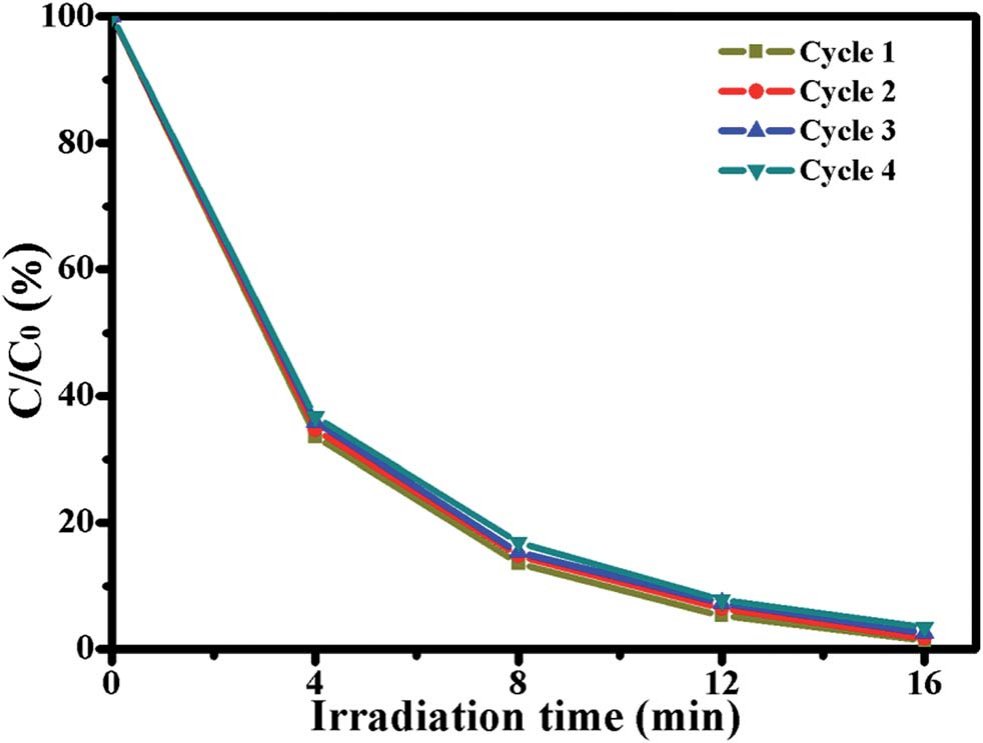
Four photocatalytic degradation cycles of RhB using Ag/ZnO under UV light.

In order to evaluate the photocatalytic stability of the photocatalyst, Ag/ZnO was chosen to degrade RhB for four photocatalytic degradation cycles under UV light. The results are shown in Fig. 8. After four photocatalytic degradation cycles, the photocatalytic efficiency of Ag/ZnO reduces by just 2.16%; Ag/ZnO exhibits high photocatalytic stability. The slight reduction of the photocatalytic efficiency is probably caused by the inevitable loss of the catalyst during the washing process.

**Fig. 9:**
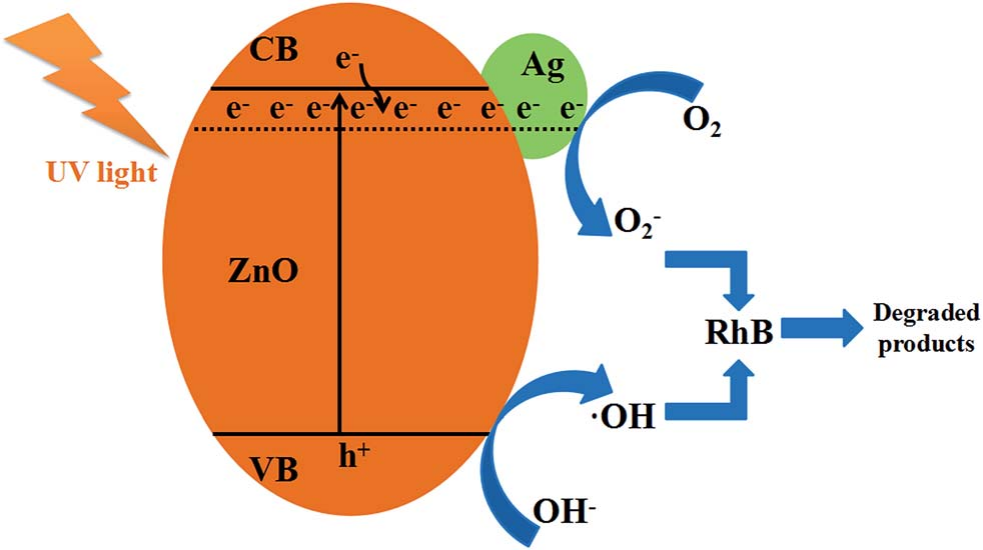
The schematic illustration of RhB degradation by Ag/ZnO under UV light.

Fig. 9 shows the schematic diagram for the photocatalytic degradation of RhB on Ag/ZnO. The band gap of ZnO is 3.20 eV. When ZnO is doped with Ag, it has more defects on its grain boundary. Ag/ZnO can be excited by light with a wavelength of less than 400 nm, promoting the valence band electrons up to the conduction band and leaving holes in the valence band as shown in eqn (2).^20,21^ The holes, produced in the valence band, can adsorb the electrons from OH^−^ or water molecules to form oxidants such as hydroxyl radicals (˙OH), as shown in eqn (3). The electrons, left in the conduction band, can combine with the dissolved oxygen to form O_2_^−^, as shown in eqn (4). ˙OH and O_2_^−^ have strong oxidability and can mineralize RhB molecules, as shown in eqn (5).

2 Ag/ZnO + *hν* → Ag/ZnO(e^−^ + h^+^)

3 h^+^ + OH^−^ → ˙OH

4 e^−^ + O_2_ → O_2_^−^

5 ˙OH + O_2_^−^ + RhB → degraded products

## 4. Conclusions

In summary, Ag doped ZnO, with enhanced photocatalytic activity, was fabricated from a ZIF-8 precursor through an adsorption method and sintering method. Ag was well loaded onto ZnO, which was prepared from ZIF-8. The activity has been successfully improved compared to that of ZIF-8, as the absorption peak of ZnO was expanded into the red wavelength region, and the photocatalytic efficiency of ZnO was increased from 92.32% to 99.64%. Furthermore, Ag/ZnO exhibited high photocatalytic stability. After four photocatalytic degradation cycles, the photocatalytic efficiency of Ag/ZnO was highly retained at 97.48%. The excellent properties of Ag/ZnO make it possible for it to be used for the degradation of organic dyes.

## Conflicts of interest

There are no conflicts to declare.

## Supplementary Material

## References

[cit1] Yang X. B., Huang L. Q., Du G. Q., Lu X. T. (2017). J. Porous Mater..

[cit2] Low J. X., Cheng B., Yu J. G. (2017). Appl. Surf. Sci..

[cit3] Maeda K., Domen K. (2016). Bull. Chem. Soc. Jpn..

[cit4] Guo X. (2004). Chem. Mater..

[cit5] Yang X. B., Chen J., Lai H. X., Hu J. P., Fang M., Luo X. T. (2017). RSC Adv..

[cit6] Ma J. F., Wang K., Li L. Y., Zhang T. L., Kong Y., Komameni S. (2015). Ceram. Int..

[cit7] Ullah R., Dutta J. (2008). J. Hazard. Mater..

[cit8] Xu C., Cao L. X., Su G., Liu W., Qu X. F., Yu Y. Q. (2010). J. Alloys Compd..

[cit9] Divband B., Khatamian M., Eslamian G. R. K., Darbandi M. (2013). Appl. Surf. Sci..

[cit10] Torad N. L., Hu M., Ishihara S., Sukegawa H., Belik A. A., Imura M., Ariga K., Sakka Y., Yamauchi Y. (2014). Small.

[cit11] Ahmad M., Ahmed E., Hong Z. L., Xu J. F., Khalid N. R., Elhissi A., Ahmed W. (2013). Appl. Surf. Sci..

[cit12] Chen Y. Q., Li J. T., Yue G. H., Luo X. T. (2017). Nano-Micro Lett..

[cit13] Jiang H. L., Liu B., Akita T., Haruta M., Sakurai H., Xu Q. (2009). J. Am. Chem. Soc..

[cit14] Nguyen L. T. L., Le K. K. A., Phan N. T. S. (2012). Chin. J. Catal..

[cit15] Kuo C. H., Tang Y., Chou L. Y., Sneed B. T., Brodsky C. N., Zhao Z. P., Tsung C. K. (2012). J. Am. Chem. Soc..

[cit16] Wee L. H., Janssens N., Sree S. P., Wiktor C., Gobechiya E., Fischer R. A., Kirschhock C. E. A., Martens J. A. (2014). Nanoscale.

[cit17] Ghosh S., Goudar V. S., Padmalekha K. G., Bhat S. V., Indi S. S., Vasan H. N. (2012). RSC Adv..

[cit18] Whang T. J., Hsieh M. T., Chen H. H. (2012). Appl. Surf. Sci..

[cit19] Pan Y. C., Liu Y. Y., Zeng G. F., Zhao L., Lai Z. P. (2011). Chem. Commun..

[cit20] Chen Y. Q., Li J. T., Yue G. H., Luo X. T. (2017). Nano-Micro Lett..

[cit21] Yang X. B., Chen J., Lai H. X., Hu J. P., Fang M., Luo X. T. (2017). RSC Adv..

